# Cyclopædia of the Practice of Medicine

**Published:** 1877-11

**Authors:** 


CYCLOPÆDIA OF THE PRACTICE OF MEDICINE--Edited by Dr. H. Von Ziemssen, Professor of Clinical Medicine in Munich, Bavaria. Vol. xii.

The twelfth volume of this library of medicine treats of diseases of the brain, and its membranes.

The five authors of the matter contained in the volume contribute as follows:

*Nothnagel*—230 pages, on Anæmia, Hyperæmia, Hemorrhage, Thrombosis and Embolism of the Brain.

*Obernier*—28 pages, on Tumors of the Brain, and its membranes.

*Heubner*—82 pages, on Syphilis of the brain and nervous system.

*Huguenin*—447 pages, on Acute and Chronic Inflammations of the Brain and its membranes.

*Hitzig*—60 pages, on Hypertrophy and Atrophy of the Brain.

